# Super-resolution microscopy to study membrane nanodomains and transport mechanisms in the plasma membrane

**DOI:** 10.3389/fmolb.2024.1455153

**Published:** 2024-09-03

**Authors:** Yenisleidy de las Mercedes Zulueta Diaz, Eva C. Arnspang

**Affiliations:** Department of Green Technology, SDU Biotechnology, University of Southern Denmark, Odense, Denmark

**Keywords:** biological membranes, super-resolution microscopy, nanodomains, membrane receptors, bioimaging data analysis, live cell imaging

## Abstract

Biological membranes are complex, heterogeneous, and dynamic systems that play roles in the compartmentalization and protection of cells from the environment. It is still a challenge to elucidate kinetics and real-time transport routes for molecules through biological membranes in live cells. Currently, by developing and employing super-resolution microscopy; increasing evidence indicates channels and transporter nano-organization and dynamics within membranes play an important role in these regulatory mechanisms. Here we review recent advances and discuss the major advantages and disadvantages of using super-resolution microscopy to investigate protein organization and transport within plasma membranes.

## Graphical Abstract

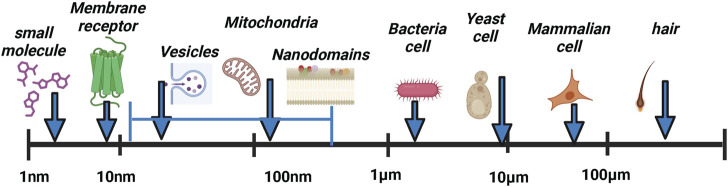

## 1 Introduction

The mammalian plasma membrane (PM) is a complex assembly of lipids and proteins that separates the cell’s interior from the outside environment (Ingolfsson et al., 2014). The multiple collective processes that take place within membranes have a strong impact not only on the cellular behavior but also on its biochemistry. Understanding these processes poses a challenge due to the often complex and multiple interactions among membrane components (Stone et al., 2017). Moreover, the PM surface accommodates different types of lipid and protein clusters (Saka et al., 2014; Owen et al., 2012; Sezgin, 2017), even though the functional role of the clustering on the membrane surface has not yet been fully understood.

Plasma membrane receptors are among the most crucial and commonly studied cell components. Not only do they ensure communication between the extracellular space and cells, but they are also responsible for regulating the cell cycle and cell division. Due to their significance, receptors have become important target structures for various pathological conditions, including Type I and II diabetes mellitus, premature ovarian failure as well as cell proliferation during cancer (Klinge and Rao, 2008). The rapid progress of our understanding of cell membrane receptors will undoubtedly open up valuable diagnostic and therapeutic possibilities for managing reproductive and endocrine disorders.

Receptors are specialized protein complexes. Being signal transducers and receivers, typically triggered upon binding of a ligand, they lead to a signaling cascade with cell response (Heldin et al., 2016). Receptors can be divided into intracellular and cell surface receptors (Alberts et al., 2015). While intracellular receptors are located entirely inside the cell, plasma membrane receptors consist of an intracellular domain, a hydrophobic membrane-spanning region, and an external ligand-binding domain, allowing communication between the extracellular space and the cells through messenger molecules (Alberts et al., 2015). Cell surface receptors can be divided into three general categories: ion channel-linked receptors (Traynelis et al., 2010), G-protein-coupled receptors (Hilger et al., 2018), and enzyme-linked receptors (Luo et al., 2019).

Plasma membrane receptors and their interactions are major drug targets because of their central roles in regulating cell communication and signal transduction (Wang et al., 2018). Despite this, knowledge about membrane receptors remains limited due to the shortcomings of the techniques available when studying the receptors in their native context. A fundamental challenge in the field of membrane receptor biology has been to investigate spatiotemporal interactions between a ligand and a receptor in the PM. This has so far been hampered by the complex structure of the cell membrane (Cao et al., 2021). This signaling-driven interaction in the PM involves not only receptors and ligands, but also membrane lipids, the underlying actin cytoskeleton, and lipid-protein crosstalk. A deeper understanding of membrane receptors has recently been accelerated by current advances in super-resolution fluorescence microscopy (SRFM) techniques which have overcome the spatial diffraction limit and thereby allowed for an unprecedented sensitive visualization and quantification of cell processes with high temporal and spatial resolution in living cells.

Intracellular transport, involving the movement of molecules, organelles, and vesicles within cells, is crucial for cellular organization, signaling, and understanding disease. Super-resolution microscopy (SRM) has transformed this field by allowing visualization of previously unresolvable molecular events, offering detailed insights into the spatial and temporal dynamics of these processes (Chen et al., 2023a; Zhang et al., 2021). Super-resolution microscopy has provided several key insights into intracellular transport: a) vesicle dynamics and cargo sorting: SRM has revealed the fine details of vesicle formation, cargo loading, and sorting within the Golgi apparatus and endosomes, showing how specific proteins are sorted into vesicles for targeted delivery (Bálint et al., 2013; Shimizu et al., 2021; Rodriguez-Gallardo et al., 2021). b) organelle interactions and contact sites: SRM has identified and characterized membrane contact sites (MCS) between organelles, such as the ER and mitochondria, which are critical for lipid exchange, calcium signaling, and coordinating transport processes (Nieto-Garai et al., 2022; Cardoen et al., 2024; Xue et al., 2020). c) motor protein function: Visualization of motor proteins moving along cytoskeletal tracks has provided insights into their stepwise movement, coordination, and cargo-binding mechanisms, contributing to a better understanding of transport efficiency and regulation (Deguchi et al., 2023; Tholen et al., 2023). d) pathogen-host interactions: SRM has been used to study how viruses and bacteria exploit intracellular transport machinery to enter cells and move to replication sites, shedding light on potential therapeutic targets (Huanhuan et al., 2024; Singh and Kenney, 2024). e) neurodegenerative disease mechanisms: In neurons, SRM has enabled the observation of axonal transport mechanisms at a resolution that reveals how disruptions in these processes contribute to diseases like Alzheimer’s and Huntington’s (Werner et al., 2021; Fuhrmann et al., 2022).

The integration of intracellular transport research with super-resolution microscopy has profoundly enhanced our understanding of cellular dynamics at a molecular level. By revealing details of vesicle trafficking, organelle interactions, and motor protein function, SRM is providing new insights into the fundamental processes that underpin cellular function and disease. This combination of technologies continues to push the boundaries of what we can observe within living cells, opening new avenues for research and therapeutic development.

This review aims to give an update on the latest advances in the knowledge of the transport within the PM in living cells, and how membrane organization might be implicated in its homeostasis, mainly by using Single-molecule localization techniques. We begin by discussing the structure and lateral organization of the cell membrane, followed by general mechanism of intracellular transport. Next, we address current challenges in the study of lipid and/or protein clusters in membranes and membrane receptors in living cells. Moreover, we, highlight how super-resolution techniques and data image analysis have contributed to expanding our knowledge, or sometimes even changing the paradigm on the structure and function of these clusters and their regulatory role in transport in the PM. Overall, significant advances have been made in the understanding of the lateral organization and transport in the PM at the nanoscale level, and some of the most relevant findings are discussed here.

## 2 The structure and organization of the cell membrane

Today, we understand that biological membranes are composed of lipid bilayers as their basic structural unit. These bilayers form the boundaries between the intracellular cytoplasm and the external environment of the cell, as well as between the interior of many of the cellular organelles and their surrounding cytoplasm. This lipid bilayer structure was first recognized as the basis of cell membrane architecture in 1925 in a detailed study of lipids (Gorter and Grendel, 1925). In 1972, Singer and Nicolson proposed the fluid mosaic model to explain the structure of the membrane (Singer and Nicolson, 1972; Chiranjeevi et al., 2009).

The Singer and Nicolson model proposes that membranes are made up of a phospholipid bilayer in which cholesterol, proteins, and carbohydrates are embedded, giving the membrane its fluid character (Singer and Nicolson, 1972). This structural composition allows the membrane to perform multiple functions such as molecular recognition, enzymatic catalysis, cell adhesion, and membrane fusion. One of the most significant aspects of this model is the introduction of dynamic features in membranes such as the diffusion of molecules in the plane of the membrane and between hemilayers (flip-flop). These important properties had not been considered in previous models. Additionally, the Singer and Nicolson model provided a more accurate description of membrane-associated proteins (Mouritsen and Bagatolli, 2016). However, its main limitation was that it gave little significance to the extensive chemical diversity of lipids observed in cellular systems.

**FIGURE 1:**
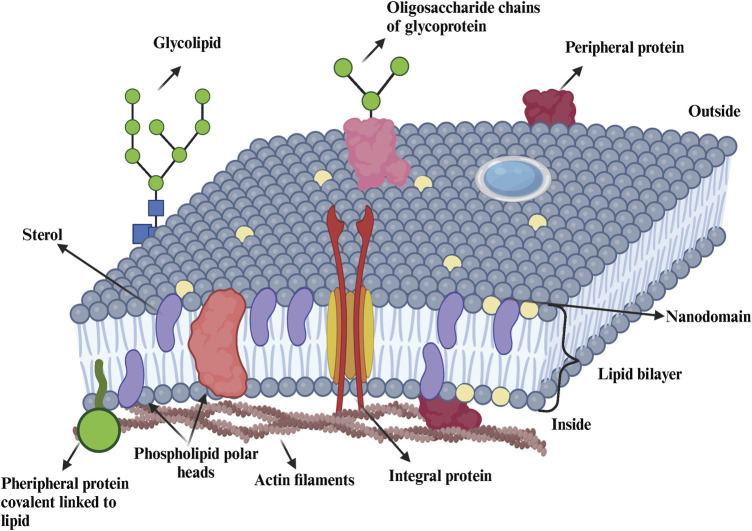
Representation of the cellular membrane. Biological membranes contain lipids (e g., sterols, phospholipids, sphingolipids), proteins (e g., integral, peripherical) and carbohydrates (glycolipids, glycoproteins). Figure is adapted from (Nelson et al., 2016; Dominguez Pardo thesis 2018).

Refinements of the fluid mosaic model have emerged over the years, generally inspired by new experimental observations or theoretical calculations that focus on specific aspect of membranes. An example is the model of J. Israelachvili, which considers the need for membrane proteins and surrounding lipids to adjust geometrically to each other (Bloom and Mouritsen, 1995). This model also incorporates the concepts of membrane folding, pore formation, and membrane thickness variations, as well as some degree of lateral heterogeneity. According to this model, lateral heterogeneity in plasma membrane refers to the lipids, proteins, and other molecules across the membrane’s surface. This heterogeneity is driven by various molecular interactions and physical properties, leading to the formation of microdomains and dynamic regions that play crucial roles in membrane function and cellular processes. Another refinement of the Singer and Nicolson model is the one proposed by E. Sackmann in 1995 (Mouritsen and Bagatolli, 2016; Bloom and Mouritsen, 1995) which emphasizes the importance of interactions between the membrane, the cytoskeleton and cell glycocalyx. The most important evolution of this model occurred in 1997 with the work of Simons and Brown (Simons and Ikonen, 1997; Brown and London, 1998). These researchers proposed that those membrane lipids organize into phase-separated microdomains, known as lipid rafts, with a composition and local dynamics distinct from the surrounding liquid crystalline phase (Stillwell, 2013). The validity of this hypothesis has been controversial and debated for more than a decade, but since then, many researchers have developed technologies to detect lateral heterogeneity in biological membranes (Carquin et al., 2016). The fluid mosaic model and the schematic of a cell membrane are illustrated in Figure 1, highlighting the complexity of the membrane´s composition.

An example of a later definition that emphasizes the dynamic nature of the domains and defines rafts as small (10–200 nm), heterogeneous, highly dynamic, sterol and sphingolipids-enriched domains that compartmentalize cellular processes. Those small rafts can sometimes be stabilized to form larger platforms through protein-protein or protein-lipid interactions. Relevant examples of the nano- and micro-organization of transmembrane proteins in clusters can be found in the work of the M. Weiss group, which shows that the axes of transmembrane proteins align approximately perpendicular to the membrane plane. The bilipid layer adapts to these proteins, leading to the formation of clusters (Morozova et al., 2011; Guigas and Weiss, 2015; Schmidt et al., 2008; Guigas and Weiss, 2008; Schmidt and Weiss, 2010).

Membranes are complex and dynamic structures that contain a wide variety of lipid species and very high protein density involved in their composition (Takamori et al., 2006; Dupuy and Engelman, 2008; Owen and Gaus, 2013). Overall, the lateral distribution of these components is likely highly non-uniform (Lingwood et al., 2008). PM is close to a miscibility critical point, and large-scale phase separation can occur near-physiological conditions (Kaiser et al., 2009). Therefore, membranes should be considered as a “lipid-protein composite” where the high density of transmembrane domains may impose order on nearby lipids, complementing lipid domains that organize proteins (Corradi et al., 2018).

Recent advances in imaging of plasma membrane proteins have revealed that transporter regulation can occur via changes in protein-protein interactions and regulated incorporation into PM microdomains (Douglass and Vale, 2005; Sako and Kusumi, 1994; Marlar et al., 2014). Such regulation can likely alter the diffusion mode of a protein, which can be measured as changes in the diffusion coefficient (Takamori et al., 2006; Douglass and Vale, 2005; Sako and Kusumi, 1994; Marlar et al., 2014). Several membrane proteins are organized in membrane nano and microdomains (Stone et al., 2017; Sezgin, 2017; Arnspang et al., 2019; Balla et al., 2012). This suggest that membrane proteins may generally organize into nanodomains, which can be regulated by various cellular stimuli.

It has also been proposed that interactions with cytoskeleton mesh or lipid domains could impose membrane receptor confinement in the PM. Some studies have reported that the actin meshwork can affect the organization of nano-domain and create barriers that might function as boundaries for the nano-domain formation in the mammalian PM (Eggeling et al., 2009; Kusumi et al., 1993). Changes in diffusion coefficients and confinement radius may be due to changes in such barriers, protein-protein interactions, proteins-lipid interactions, or other conformational changes.

Overall, three models could explain molecular dynamics and domain structure in PM. These models involving: 1) temporary binding and/or transient entrapment within nanoscale domains (Eggeling et al., 2009; Sahl et al., 2010; Honigmann et al., 2014), 2) free diffusion (Brownian motion) (Gambin et al., 2006; Frick et al., 2007), and 3) link to cytoskeleton protein as pickets and fences (Eggeling et al., 2009). However, a model that has been reported in several mammalian cells is the transient confinement within a compartment and hop diffusion of molecules in PM. Hence, trajectories can be described by two distinct diffusion coefficients, depending on the considered spatio-temporal scale: a microscopic one that describes the free diffusion of molecules within the nanodomains and a macroscopic one that describes their slower effective diffusion across the plasma membrane (Cooper, 2000). In this approach, the molecules undergoing Brownian diffusion are interrupted by frequent transient entrapment and consider the PM molecular events occurring on a greater scale (transmembrane proteins undergo macroscopic diffusion).

The introduction of advanced biophysical techniques (single-particle tracking, fluorescence correlation spectroscopy (FCS), fluorescence recovery after photobleaching) has provided new arguments for the existence of isolated membrane domains of nanometric size, enriched with cholesterol and sphingolipids, and of a highly dynamic nature (Mouritsen and Bagatolli, 2016; Kusumi et al., 1993). The major advantage of these approaches is that they can be performed on living cells in physiological conditions, minimizing disruptions of cellular systems. Additionally, in the past decade, super-resolution techniques and image analysis have allowed us to obtain highly quantitative data about complex spatio-temporal dynamics and distribution of membrane receptors at the nanoscale level. Further details and the latest advances in this topic are discussed in Section 2.1 of this review.

### 2.1 Super-resolution microscopy in mammalian cells to study membrane nanodomain organization and dynamics and their impact on the transport in the PM

Understanding the complex scenario where membrane components interact at the nanoscale and also impact cellular behavior, poses a major challenge (Sezgin, 2017). Cellular membranes are hubs for cellular signaling. They are heterogeneous structures accommodating clusters, domains, and nano assemblies, and this heterogeneity is crucial for cellular signaling (Barbotin et al., 2020). Associated with these structures are active dynamic cellular processes with timescales ranging from a cell division that occurs over several minutes to fast diffusion exhibited by membrane lipids on millisecond timescales. In this context, and noteworthily, time-lapse images of interesting processes such as cell division, and mitochondrial fission/fusion (Hailey et al., 2010; Terasaki, 1995) have been successfully captured using conventional fluorescence microscopy techniques, providing valuable insight into these processes. However, understanding mechanisms such as protein clustering and diffusing in the PM due to signal transduction, and actin polymerization at higher resolution has highlighted the need for a technique with higher spatiotemporal resolution (Roobala et al., 2018). Therefore, there has been an extensive effort to resolve the mystery of nanoscale structure and dynamics of cellular membranes (Barbotin et al., 2020).

SRFM techniques will be key to moving fluorescence microscopy and bioimaging areas forward. With advancements in the field of optical nanoscopy, new light has been shed on intricate details of the cellular machinery, unraveling spatial and dynamic processes from different aspects of the cell functioning (Blom and Bates, 2015; Sahl et al., 2010). Super-resolution can be used to increase both axial and lateral resolution, achieving resolutions down to tens of nanometers and, with more recent technologies such as Minimal Photon Fluxes, even 1 nm. However, additional effort is needed to improve temporal resolution to capture fast cellular processes and expand the use of these techniques in live-cell applications.

### 2.2 Super-resolution techniques

**FIGURE 2:**
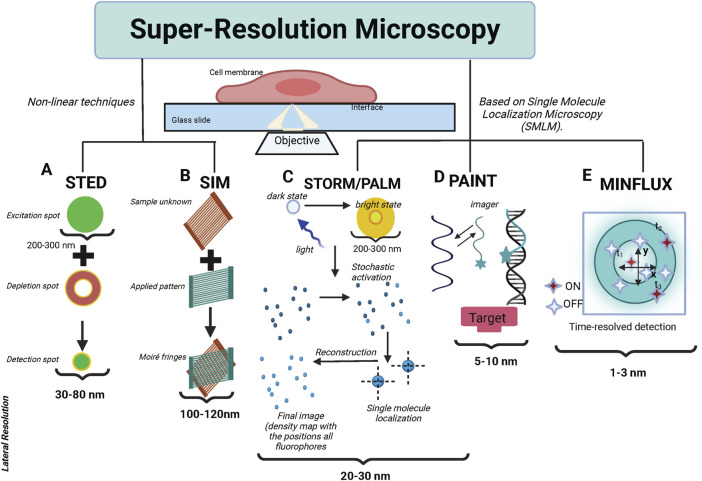
The fundamental principles of the primary super-resolution techniques. **(A)** In STED, the sample plane is concurrently illuminated by an excitation and a depletion beam. The depletion beam, with a donut-shaped profile, selectively depletes most of the excited molecules nonlinearly into the dark state via stimulated emission, preserving the center of the excitation PSF. Consequently, this results in an effective PSF, smaller than the original diffraction-limited PSF. The sample is then scanned with this effective PSF to generate a super-resolution image. **(B)** In SIM, the sample is illuminated with a pattern of light and the resulting fluorescence signal is collected. By analyzing the interference pattern between the illumination pattern and the sample structure, high-resolution information is extracted. This allows for the reconstruction of a super-resolved image with a resolution approximately twice that of conventional light microscopy. **(C)** STORM (Stochastic Optical Reconstruction Microscopy) and PALM (Photo-Activated Localization Microscopy), fluorescent probes are activated and imaged sequentially, with only a small subset of probes being active at any given time. By precisely localizing individual fluorescent molecules, a super-resolution image can be reconstructed with a resolution far beyond the diffraction limit. **(D)** DNA PAINT (Point Accumulation for Imaging in Nanoscale Topography utilizes transient binding between complementary DNA strands for high-resolution imaging. It employs fluorescently labeled docking strands attached to the target structure, and complementary imaging strands with a fluorescent dye in the imaging solution. The imaging strands transiently bind to the docking strands, allowing the fluorescence signals from individual molecules to be detected and localized. This repeated imaging and localization of individual molecules reconstruct a super-resolved image of the target structure beyond the diffraction limit. **(E)** MINFLUX utilizes a doughnut-shaped excitation pattern, with a minimum in the center. The position of the minimum is adjusted to center around the fluorophore to be localized, allowing precise determination of its location. MINFLUX achieves extremely high spatial resolution by narrowing down the position of a fluorophore to a small region, surpassing the diffraction limit.

The two main types of super-resolution microscopy techniques: The first is based on nonlinear techniques including Stimulated Emission Depletion (STED) microscopy (Willig et al., 2006) and Structured-Illumination Microscopy (SIM) (Gustafsson, 2005). The second is based on single-molecule localization including Stochastic Optical Reconstruction Microscopy (STORM) (Rust et al., 2006)/Photoactivated Localization Microscopy (PALM) (Betzig et al., 2006) and Point Accumulation for Imaging in Nanoscale Topography (PAINT) microscopy (Sharonov A. and Hochstrasser R. M., 2006). More recently, Minimal Photon Fluxes (MINFLUX) (Balzarotti et al., 2017) have been developed, this concept pushed the resolution to 1–3 nm in three dimensions. These are called SMLM (Single-molecule localization microscopy), and the principles of these methods are illustrated in Figure 2. Table 1 provides a comparative summary of the advantages and disadvantages of various SMLM techniques in membrane research.

**TABLE 1:** A comparative table summarizing the advantages and disadvantages of different single-molecule localization microscopy (SMLM) techniques in the context of membrane studies is provided below.

SMLM technique	Advantages	Disadvantages
PALM (Photoactivated Localization Microscopy)	- High spatial resolution (20–30 nm) - Suitable for live-cell imaging, enabling real-time study of membrane dynamics - High specificity with photoactivatable fluorescent proteins	- Phototoxicity due to repeated photoactivation - Photobleaching limits imaging duration - Complex optical setup and data processing
STORM (Stochastic Optical Reconstruction Microscopy)	- Extremely high resolution (10–20 nm) - Capable of multiplexing for simultaneous imaging of different membrane components - Uses photostable organic dyes, allowing for long-term imaging	- Slow acquisition time, limiting live-cell imaging - Harsh sample preparation conditions may affect native membrane structures - Computationally intensive data processing and reconstruction
PAINT (Points Accumulation for Imaging in Nanoscale Topography)	- High resolution comparable to STORM - Minimal photobleaching, ideal for extended imaging sessions - Simple sample preparation, preserving native membrane environment	- Slower imaging due to reliance on accumulation of binding events - Less suitable for live-cell imaging - Limited by the availability of suitable probes for specific membrane targets

Overall, single-molecule signals can be identified by their shape, size, expected fluorescent intensity as well as by their quantal “blinking” behavior. Mathematical fitting, localizing the centroid of these PSFs defines the precise xy coordinates from where the signals arose; the certainty of localization is affected by brightness, noise, and pixel size. By rendering coordinates, a virtual image is created which shows the coordinate positions of all the molecules on the plasma membrane that have emitted. In general, SMLM methods usually employ conventional wide-field excitation and achieve super-resolution by localizing individual molecules (Lelek et al., 2021; Heilemann et al., 2002; Fölling et al., 2008; Schnitzbauer et al., 2017; Manley et al., 2008; Bintu et al., 2018; Weisenburger et al., 2017; Klein et al., 2014; Van de Linde et al., 2011).

In STED nanoscopy, a nanoscale resolution is achieved by de-exciting, excited molecules to the ground state in a nonradiative manner using a depletion laser, as shown in schematics (Figure 2). Vortex phase filters are used to create a donut-shaped beam. This phase filter is designed to ensure the canceling of depletion beam intensity in the center through destructive interference (Möckl and Moerner, 2020). In STED nanoscopy, a “donut-shaped beam” is essential for achieving super-resolution imaging. After a standard laser excites fluorophores in a sample, a second, donut-shaped beam is applied. This beam depletes fluorescence in all regions except for a tiny central spot, improving resolution beyond the diffraction limit. The beam’s dark center, created through destructive interference, ensures minimal light intensity at the center, allowing only nearby fluorophores to emit fluorescence. This interference is intentionally engineered to occur at the beam’s center, creating a region of minimal or no light. By carefully controlling the phase and amplitude of the light waves that form the donut shape, the area of destructive interference can be precisely manipulated, further refining the microscope’s resolution. This selective depletion sharpens the imaging area, enabling visualization at the nanometer scale. This technique works exactly like confocal scanning where the scanning speeds are high but with a sub-diffraction spot. STED can be easily used for live cell tracking, video nanoscopy, or combined with techniques such as FCS, which offer the best time resolution down to a few microseconds (Hell, 2007; Hell et al., 2015; Sezgin, 2017). This makes STED advantageous over other super-resolution techniques in capturing the pathway of dynamical cellular processes with nanoscale resolution. However, a limitation of STED microscopy is the use of high laser intensities, potentially introducing high phototoxicity and photobleaching, which usually prevent imaging of living cells over long periods.

STED has been used to examine Ca^2+^ channel clustering induced by the exocytotic machinery (Kittel et al., 2006) as well as probing the nanoscale structure of the synapse (Hiersemenzel et al., 2013; Opazo et al., 2010; Westphal et al., 2008; Roobala et al., 2018). It has been also used to study the nature and origin of membrane heterogeneities at the nanoscale in live cells and model membranes (Honigmann et al., 2014; Sezgin, 2017), the role of exocytosis and endocytosis in the synapses (Willig et al., 2006), dynamics of certain organelles such as Golgi bodies and endoplasmic reticulum (Hein et al., 2008). All of which, would not have been solved by using confocal imaging.

STED can be combined with spectroscopic tools to probe the physical and chemical properties of membranes with nanoscale resolution (Sandhoff et al., 2009; Schneider et al., 2017; Sezgin, 2017). The combination of STED combined with fluorescence correlation spectroscopy FCS (STED-FCS) has been used extensively to address the nanoscale membrane structure (Benda et al., 2015; Hedde et al., 2013; Schneider et al., 2018; Vicidomini et al., 2015) and dynamics in a wide range of processes in cells. STED-FCS is a sensitive tool for studying nanoscale membrane organization, as well as providing details about molecular membrane dynamics, such as lipid-protein interactions, which may play an important role in cellular functionalities (Mueller et al., 2011). To capture fast dynamics, scanning STED-FCS, circular scanning STED-FCS, raster imaging correlation spectroscopy (RICS) (Lelek et al., 2021) STED and cross-pair correlation spectroscopy (pCF) STED has been employed (Bianchini et al., 2014). Recently, STED has also been combined with spectral imaging and polarity-sensitive probes to quantitatively study the nanoscale physiochemical properties of the membrane (Barbotin et al., 2020).

An alternative to the super-resolution approach is Structured-illumination microscopy (SIM). In SIM, the fringe patterns of different orientations and phase shifts are used to illuminate samples. These patterns interact with the fine details of the sample, creating moiré patterns—interference patterns that occur when two sets of periodic structures overlap. Moiré patterns shift high-frequency information, normally beyond the diffraction limit, into the observable range of the microscope, allowing the capture of details that are otherwise too small to be seen. Multiple images are taken with the illumination pattern shifted in different orientations and phases. These images are then computationally reconstructed to extract the high-frequency information encoded in the moiré patterns. By analyzing these patterns, SIM can double the resolution of a conventional microscope, enabling visualization of structures at around 100 nm, compared to the usual 200–300 nm limit. Compared with STED and SMLM, SIM has several excellent characteristics, such as simple sample preparation, fast imaging speed, and minimally invasive. Those characteristics made it suitable for capturing the fast dynamics of live samples (Kaufmann et al., 2011; Lemmer et al., 2008). SIM lies in its low photo-toxicity to bio-samples, which enables imaging of live samples for a longer time. Considering several raw images are needed to reconstruct one super-resolved SIM image, SIM is still limited to samples with slower movements. Despite being already powerful, SIM still needs further improvements to make it more functional and useful for complex biological problems in the future (Ma et al., 2021; Richter et al., 2020).

Jonathon et al. have applied high-speed SIM and successfully captured the fast dynamics of organelles inside the live cells with a lateral resolution of ∼97 nm, as well as an imaging depth of ∼1 μm above the sample surface (Nixon et al., 2016). In addition, Guo et al., proposed multi-color grazing incidence illumination (multi-color GI-SIM), and utilized it to investigate several important biological phenomena, such as ER-branching events (Guo et al., 2018). This investigation found that tubular ER plays a vital role in mitochondrial fusion and fission, transporting and controlling the local concentration of intracellular organelles, such as late endosomes or lysosomes. Moreover, applying live cell confocal and Structured Illumination (SR-SIM) super-resolution imaging, showed that B cells respond to bacterial (Cholera) toxin challenge by their subsequent internalization followed by rapid formation of intracellular microvesicles (MVs). This result highlights the power of super-resolution microscopy to uncover so far hidden structural details of biological processes, such as microvesicle formation and transport (Halász et al., 2018).

SMLM techniques such as PALM/STORM have circumvented the diffraction limit of resolution to enable the localization of single molecule point-spread function (PFS) signals that are separated in time as shown in Figure 2. Cycles of activation, followed by imaging and photo destruction are repeated over and over again acquiring 1,000s of images in total. The core idea behind SMLM is to chemically or physically govern molecules (fluorescent dyes or proteins) in such a way that in each frame only a small fraction of molecules emit fluorescence signals (Henriques et al., 2011). The lateral location of these sparsely distributed molecules is then determined by Gaussian peak fitting with high precision to perform super-resolution. In order to reconstruct a super-resolution image with a typical resolution of around tens of nanometers, SMLM always needs to record thousands of images. SMLM can be implemented with organic dyes, e.g. photoswitchable Cy3–Cy5 dye pairs in stochastic optical reconstruction microscopy (STORM) (Blom and Bates, 2015; Henriques et al., 2011). Meanwhile, analogous methods have been proposed and implemented by using fluorescent proteins to achieve the lateral resolution of ∼20 nm. For instance, a photoconvertible fluorescent protein named EosFP in photoactivated localization microscopy (PALM) has been explored (Nifosì et al., 2024; Nienhausa and Nienhaus, 2014; Patterson et al., 2010; Shcherbakova et al., 2014). In our team, we have recently conducted some experiments by using PALM and PALM combined with Expansion Microscopy, as powerful tools to study the organization and dynamics of the Angiotensin II type 1 receptor, AT1R with nanometer resolution in a cellular context. From these results, we concluded that AT1R lateral diffusion increased after binding to Angiotensin II (Ang II), and the receptor diffusion was transiently confined in the PM (Andersen et al., 2022).

A variation of PALM combines single-particle tracking, so-called sptPALM, allowing the high-precision tracking of thousands of single molecule signals with high temporal resolution. In this context, it should be noted that even super-resolution techniques such as PALM and STORM, which need elaborate data processing to reconstruct images with high spatial resolution, fail to capture fast dynamical processes (Betzing et al., 1991; Moerner, 2015) However, recently, video-rate STORM has been shown to record the motion of transferrin receptors in the plasma membrane. PALM is already starting to be used to define ion channel molecule localization, recently employed to image single aquaporin molecules (Opazo et al., 2010). Other applications of SMLM with clinical relevance are discussed in the following references (Boyd et al., 2016; Pilarczyk et al., 2017; Bartosova et al., 2021; Bartosova et al., 2020).

PALM and STORM have contributed significantly to the comprehension of biological complexes. Nevertheless, both methods suffer from the limitation of providing only a restricted number of observations for each labeled molecule. This limitation is attributed to the constraints imposed by the photon budget of a fluorophore, which refers to the maximum number of photons a fluorophore can emit before undergoing bleaching. Consequently, this under-sampling of the true underlying biology occurs. Techniques that enable multiple repeated observations of molecules, without being constrained by the photon budget, pose a solution for super-resolution microscopy. An example of such a technique is point accumulation for imaging in nanoscale topography (PAINT) (Sharonov A. and Hochstrasser R. M., 2006).

DNA-PAINT is a type of single-molecule localization microscopy. It involves the transient binding of dye-labeled DNA strands to complementary docking strands attached to the target molecules. The binding and unbinding events are detected, and the localization information is used to reconstruct super-resolved images. As the kinetics of the probe ensure unbinding, the fluorescent signal is turned off again until a new molecule binds. As the probes are replenished continuously, it is not sensitive to photobleaching, which is a major advantage of this method over other SMLM techniques. This allows for long imaging times and thus a higher accuracy. Moreover, multiplexing can be achieved by combining multiple probes with different dyes (Tholen et al., 2023). DNA-PAINT offers high spatial resolution, allowing for the visualization of molecular details. It can be adapted to various biological samples, making it a versatile tool for studying different cellular structures (Schnitzbauer et al., 2017).

DNA-PAINT is a super-resolution microscopy technique that allows for the visualization of biological structures at the nanoscale level. It is particularly useful for studying the plasma membrane and cellular dynamics. DNA-PAINT allows us to visualize structures in the plasma membrane at a resolution beyond the diffraction limit, providing detailed information about the arrangement of molecules at the nanoscale. It is particularly suitable for studying dynamic processes, such as the transport of molecules within the plasma membrane (Niederauer et al., 2023; Oleksiievets et al., 2022). DNA-PAINT-based single-particle tracking (DNA-PAINT-SPT) has recently significantly enhanced observation times *in vitro* SPT experiments by overcoming the constraints of fluorophore photobleaching (Niederauer et al., 2023).

In some studies, DNA-PAINT has been adapted to investigate the intracellular transport of vesicles and organelles. By attaching DNA strands to vesicle surfaces or specific organelle markers, researchers can visualize and track the movement of these structures within the cell. This application enables the study of intracellular trafficking dynamics, including the transport of vesicles along microtubules or other cytoskeletal elements (Tholen et al., 2023). It is worth noting that while DNA-PAINT offers high spatial resolution, other techniques like single-particle tracking and live-cell imaging using traditional fluorescent markers are also commonly used for transport studies. The choice of method depends on the specific research questions, the biological system under investigation, and the desired spatiotemporal resolution.

Researchers have employed DNA-PAINT to study the dynamics of membrane proteins on the cell surface. By labeling membrane proteins with DNA-conjugated antibodies or DNA-labeled ligands, researchers can track the movement and interaction of individual proteins with high spatial resolution. This approach gives insights into the diffusion properties and clustering behavior of membrane proteins, shedding light on their role in cellular processes. Motor-PAINT is a super-resolution microscopy technique that combines aspects of single-molecule localization microscopy with the controlled movement of molecular motors to achieve high-resolution imaging. The term “PAINT” stands for “Points Accumulation for Imaging in Nanoscale Topography,” and the “Motor” prefix refers to the use of motor proteins, such as kinesin or myosin, to facilitate the controlled movement of fluorescent probes. Motor-PAINT extends the principle of PAINT based on endogenous protein-protein interactions aimed to resolve the actin and microtubule cytoskeleton (Tholen et al., 2023; Oleksiievets et al., 2022) In addition, DNA-PAINT imaging has also provided a chance to observe cell surface receptors such as ryanodine receptors (RyRs) (Jayasinghe et al., 2018), epidermal growth factor receptors (EGFRs), insulin-like growth factors (IGFs) and the protein family ErbB (Werbin et al., 2017; Schröder et al. (2021) have been used DNA-labeled primary antibodies, they visualized all four FGFRs in the same cell with near-molecular spatial resolution. From the super-resolution imaging data, they extracted information on FGFR density, spatial distribution, and inner subfamily colocalization.

Further examples of DNA-PAINT applications in plasma membrane studies, nanoscale imaging, dynamics processes, or transport studies can be found in the reviews by Nieves et al. (2018), and by Tholen et al. (2023). These reviews also highlight the current challenges for DNA-PAINT imaging as we move forward.

MINimal emission FLUXes, (MINFLUX) is a super-resolution microscopy technique developed to achieve extremely high spatial resolution. MINFLUX relies on the precise localization of individual fluorophores rather than their simultaneous detection. This is achieved by sequentially turning on and off the fluorescence of molecules. The imaging process involves an iterative cycle of activation, detection, and photobleaching. A single fluorophore is activated at a time, its emission is detected, and then it is photobleached before the next cycle begins. MINFLUX utilizes a doughnut-shaped excitation pattern, with a minimum in the center. The position of the minimum is adjusted to center around the fluorophore to be localized, allowing precise determination of its location. MINFLUX achieves nanometer resolution by narrowing down the position of a fluorophore to a small region, surpassing the diffraction limit. The sequential activation and detection of fluorophores contribute to a better signal-to-noise ratio, enhancing the quality of the acquired images. One of the major advantages of this technique is that provides spatial resolution beyond the diffraction limit, allowing researchers to visualize biological structures at the nanoscale with unprecedented detail. Moreover, because MINFLUX activates and detects fluorophores sequentially, it minimizes the exposure of fluorophores to light, reducing photobleaching and phototoxic effects on the sample. MINFLUX can acquire super-resolution images relatively quickly compared to some other techniques, making it suitable for studying dynamic biological processes (Deguchi et al., 2023; Eilers et al., 2018; Schmidt et al., 2021; Pape et al., 2020; Eilers et al., 2018; Schmidt et al., 2021; Pape et al., 2020).

However, the implementation of MINFLUX microscopy requires a sophisticated setup and the technique itself can be complex, limiting its accessibility to some researchers. Achieving optimal MINFLUX performance relies on precise control of the excitation pattern, which may require specialized hardware and expertise. Furthermore, the choice of suitable fluorophores is crucial for MINFLUX, and not all fluorophores may be compatible with the technique. On the other hand, sample preparation for MINFLUX may require specific conditions, and not all samples are equally amenable to this technique. Despite these challenges, MINFLUX continues to be a powerful tool for researchers pushing the boundaries of super-resolution microscopy. Advances in technology and methodology are likely to address some of these limitations over time.

MINFLUX microscopy, with its high spatial resolution and minimal photobleaching, is valuable for studying protein dynamics at the nanoscale. Among the main applications of MINFLUX to investigate protein dynamics are: 1) Single-protein tracking: MINFLUX enables the precise localization of individual fluorophores, making it suitable for tracking the movement of single proteins within cells. By attaching fluorophores to specific proteins of interest, researchers can study their diffusion, interactions, and dynamic behavior in real time (Eilers et al., 2018; Schmidt et al., 2021; Carsten et al., 2022). 2) Membrane Protein Dynamics: MINFLUX is well-suited for studying membrane proteins, including their lateral mobility and interactions within the cell membrane. Researchers can investigate the dynamics of individual membrane proteins and their behavior in response to various cellular stimuli (Pape et al., 2020). 3) Protein Complex Formation: studying the formation and dissociation of protein complexes is crucial for understanding cellular processes. MINFLUX allows researchers to observe the dynamics of protein interactions at a high level of detail. By labeling individual proteins within a complex, researchers can track their movement and interactions over time (Pape et al., 2020; Fischer et al., 2021; Carsten et al., 2022). 4) Intracellular Transport: MINFLUX can be applied to study the dynamics of intracellular transport of proteins, vesicles, and other cellular structures. Tracking the movement of labeled proteins provides insights into the mechanisms of intracellular transport and the organization of cellular compartments (Deguchi et al., 2023; Prakash, 2022; Liu et al., 2022; Carsten et al., 2022).5) Dynamic Changes during Cellular Processes: investigating dynamic changes in protein localization and interactions during cellular processes, such as cell division or differentiation, is possible with MINFLUX. Researchers can capture high-resolution images of proteins undergoing dynamic changes, contributing to a better understanding of cellular dynamics (Bond et al., 2022; (Deguchi et al., 2023; Pape et al., 2020). 6) Protein Aggregation Studies: For proteins associated with diseases involving misfolding or aggregation, MINFLUX can be used to study the dynamics of protein aggregates. Observing the behavior of individual proteins within aggregates may provide insights into the progression of neurodegenerative diseases and other protein misfolding disorders (Schmidt et al., 2021; Simpson et al., 2020; Grabner et al., 2022). 7) Mapping Subcellular Structures: MINFLUX can be employed to map the spatial distribution and dynamics of proteins within subcellular structures. This application is particularly useful for understanding the organization of organelles and the dynamic interactions between proteins within these structures (Galiani et al., 2023; Carsten et al., 2022; Sigal et al., 2018; Ostersehlt et al., 2022).

MINFLUX offers high spatiotemporal resolution and reduced photobleaching. It undoubtedly provides a powerful tool for unraveling the intricate dynamics of proteins in living cells. Researchers keep on exploring and expanding the applications of MINFLUX for studying various aspects of protein behavior at the nanoscale.

Overall, SRFM techniques have narrowed down the resolution gap between light microscopy and Electron Microscopy (EM). They have facilitated breakthroughs in molecular cell biology, while life sciences have aimed to decipher subcellular and molecular organization at nanoscale resolution (Yang and Annaert, 2021). Emerging correlative approaches of SRFM with (cryo-)EM further combine their respective advantages, to the point of enabling 3D analysis of nanoclusters/structures *in situ* (Weisenburger et al., 2017; Bock and Grubmüller, 2022; Subramaniya et al., 2023). In addition, other combined SRFM modalities, such as lattice light-sheet microscopy combined with SMLM and various 3D super-resolution microscopy techniques, will also enhance the study of native nanoscopic structures (Wäldchen et al., 2020; Lelek et al., 2021; Gagliano et al., 2021; Liu et al., 2020; Malivert et al., 2022).

### 2.3 Super-resolution imaging and extraction of quantitative information

Super-resolution imaging, coupled with quantitative analysis, has become an indispensable tool for researchers studying the intricate details of cellular and molecular structures. The continuous development of techniques and analysis methods contributes to advancing our understanding of complex biological processes. Specialized software tools are essential for the post-processing and quantitative analysis of super-resolution images to extractmeaningful information from the data collected. These tools often include algorithms for single-molecule localization, colocalization studies, and statistical analysis (Khater et al., 2020). Key aspects regarding super-resolution imaging and quantitative information extraction are shown in the table below (see Table 2):

**TABLE 2:** Summary of the main parameters used in super-resolution image analysis and quantification methods in membrane research.

Parameter	Applications	References
Single-Molecule Tracking	Allows for the monitoring of individual protein and lipid movements within the membrane Provides data on diffusion coefficients, interaction rates, and pathway dynamics	(Chen et al., 2023b), (Hebert et al., 2005), (Sehayek et al., 2021)
Clustering Analysis	Identifies and quantifies the organization of membrane proteins into functional clusters Utilizes algorithms like DBSCAN (Density-Based Spatial Clustering of Applications with Noise) to analyze spatial patterns	(Griffié et al., 2017), (Khater et al., 2020), (Andronov et al., 2018), (Williamson et al., 2020), (Feng et al., 2022), (Pilarczyk et al., 2017)
Quantitative Co-Localization	Measures the degree of overlap between different membrane components Assesses the functional relationships and interactions between proteins and lipids	(Schnitzbauer et al., 2017), (Fischer et al., 2021), (Tameling et al., 2021), (Lew et al., 2011)
Fluorescence Intensity Analysis	Determines the concentration and distribution of specific molecules within the membrane Employs techniques like fluorescence correlation spectroscopy (FCS) for precise quantification	(Andronov et al., 2018), (Lukeš et al., 2017), (Nan et al., 2013), (Durisic et al., 2014)

Membrane research is pivotal for understanding cellular membranes’ structural and functional dynamics. These membranes play crucial roles in cellular processes, including signal transduction, molecular transport, and cell communication. Advances in imaging techniques, particularly super-resolution microscopy, have revolutionized our ability to study membranes at unprecedented levels of detail. Quantitative analysis is essential for translating super-resolution images into meaningful biological insights. The combination of super-resolution imaging and quantitative analysis has revealed how proteins and lipids interact to facilitate membrane curvature, trafficking, and signaling (Hartley et al., 2015; Caetano et al., 2015; Baranov et al., 2019). Additionally, these techniques enable the observation of real-time behavior of membrane components during processes such as endocytosis, exocytosis, and cell division (Mini et al., 2015; Zalejski et al., 2023), as well as the identification of alterations in membrane organization and function associated with diseases like cancer, neurodegeneration, and infections (Conze et al., 2022; Werner et al., 2021).

By utilizing these advanced methods, researchers can gain a deeper understanding of the complex and dynamic nature of cell membranes, paving the way for new therapeutic strategies and a greater comprehension of cellular function.

## 3 Advancing super-resolution microscopy: addressing current challenges in investigating protein organization within plasma membrane nanodomains and exploring future perspectives

Super-resolution microscopy has revolutionized the field of cell biology by enabling the visualization of cellular structures at a resolution beyond the diffraction limit of light microscopy. This breakthrough technology has provided unprecedented insights into the organization and dynamics of proteins within plasma membrane nanodomains. In this literature review, we discuss the major advantages and disadvantages of using super-resolution microscopy to investigate protein organization and transport within plasma membranes. In this section, we address the current challenges faced in investigating protein organization within plasma membranes using super-resolution microscopy and explore future perspectives for advancing this field.

One of the main obstacles in super-resolution microscopy is the limited availability of fluorophores that possess the necessary photophysical properties for achieving high-resolution imaging. Another critical aspect of super-resolution microscopy is the specific targeting of proteins of interest within plasma membrane nanodomains. This is particularly challenging due to the dynamic nature of the plasma membrane, where proteins constantly diffuse and interact with various cellular components. A common challenge in super-resolution microscopy (SRM) is balancing the need for a high number of detections with preserving the native physiological environment. Overexpressing a fluorescently tagged protein can generate abundant data, which is beneficial for achieving statistically significant results. However, this overexpression can also introduce artifacts, such as protein aggregation, mislocalization, or disruptions to cellular processes due to the unnaturally high protein concentration. In contrast, expressing the protein at endogenous levels is more biologically relevant, as it reflects natural physiological conditions and avoids the artifacts associated with overexpression. However, the downside is that endogenous expression levels may be too low to produce a sufficient signal for SRM, complicating the visualization and analysis of the protein of interest (Chen et al., 2024). To overcome the hurdle of obtaining SRM-applicable sample formats while maintaining them under their closest physiological setup, induced pluripotent stem cells (iPSCs) provide a promising option for SRM studies. iPSCs are adult cells reprogrammed to exhibit pluripotency, allowing them to differentiate into various cell types (Takahashi and Yamanaka, 2006).

Several labeling strategies have been employed to address this issue, including genetic tagging, chemical labeling, and antibody-based targeting. Genetic tagging involves the fusion of a fluorescent protein or a small peptide tag to the protein of interest, allowing for its visualization within the cell. This approach offers high specificity and compatibility with live-cell imaging, but it may alter the native behavior of the tagged protein or interfere with its localization. Chemical labeling, on the other hand, relies on the use of small molecules that can selectively bind to specific proteins or protein domains. Contreras et al., developed a cholesterol probe, chol-N3, which, when used with super-resolution microscopy, allows for the direct visualization and characterization of lipid rafts with unprecedented resolution in living cells. Chol-N3 mimics cholesterol without disrupting cellular or synthetic membranes and reveals the presence of cholesterol-rich nanodomains smaller than 50 nm in the plasma membrane of resting live cells (Lorizate et al., 2021).

Researchers have studied the L-type calcium channel CaV1.3, which is vital for voltage-dependent Ca^2^⁺-signaling in heart and brain cells, using advanced imaging techniques. By employing a HaloTag-labeling strategy and STED nanoscopy, they visualized and quantified CaV1.3 clusters in HEK293 cells. The study revealed that these clusters have lower-than-expected channel densities and that their size and density increase after treatment with isoprenaline, indicating a regulated condensation mechanism (Schwenzer et al., 2024). Another group, using STED, has achieved high temporal resolution, allowing them to observe synaptic vesicle dynamics, endocytosis, exocytosis, and endosomal interactions in real time (Alvelid et al., 2022).

This approach offers high specificity and versatility but requires the design and synthesis of suitable labeling probes for each target protein. Antibody-based targeting utilizes the high affinity and specificity of antibodies to recognize and bind to specific proteins. This approach offers excellent specificity and compatibility with fixed-cell imaging, but it requires the availability of highly specific antibodies for each target protein.

Despite the availability of various labeling strategies, challenges still exist in achieving efficient and specific protein labeling in super-resolution microscopy. One major challenge is the potential interference of the labeling strategy with the native properties and functions of the target protein. For example, genetic tagging may disrupt protein-protein interactions or alter protein folding, leading to mislocalization or functional impairment. Chemical labeling may introduce steric hindrance or alter the conformation of the target protein, affecting its biological activity. Antibody-based targeting may suffer from nonspecific binding or insufficient antibody penetration into densely packed cellular structures. Therefore, careful consideration and validation of the chosen labeling strategy are necessary to ensure accurate interpretation of the obtained super-resolution images (Valli et al., 2021).

To overcome these challenges, future research efforts should focus on the development of improved labeling strategies that minimize interference with the native properties and functions of the target proteins. This may involve the design of smaller and less perturbing tags or the development of labeling techniques that can be applied directly to endogenous proteins without the need for genetic modification. Additionally, the optimization of labeling conditions, such as buffer composition, labeling probe concentration, and incubation time, can significantly enhance labeling efficiency and specificity. Furthermore, the development of advanced imaging modalities, such as correlative super-resolution microscopy and electron microscopy, can provide complementary information about the ultrastructure and localization of labeled proteins.

Super-resolution microscopy techniques like STED and SMLM are highly sensitive to photobleaching and phototoxicity, which can lead to the loss of fluorescent signals over time and limit the duration of live-cell imaging experiments. The high-intensity laser beams used for excitation in these techniques accelerate photobleaching, which can be problematic for studying dynamic processes or samples with low fluorophore densities. To address this, researchers employ strategies such as using lower laser powers, reducing exposure time, and utilizing photostable or reversible photoswitchable fluorophores. Advanced imaging modalities, including time-lapse and multi-color imaging, also help monitor dynamic processes while minimizing photobleaching. Winkelmann et al., have combined flat-field illumination with multi-angular TIR to enable quantitative multi-color SMLM with uniform illumination. This setup, using dual cameras for up to three channels, allows for robust detection of receptor stoichiometries, smFRET analysis, and long-term tracking and localization microscopy (TALM). By integrating TALM with structured-illumination microscopy (SIM), the method correlates molecular diffusion properties with nanoscale cytoskeletal organization and dynamics (Winkelmann et al., 2024).

Phototoxicity is a major challenge in super-resolution microscopy, as intense illumination can harm biological samples, leading to cell damage, altered behavior, and even cell death. The high laser powers used in these techniques can cause photochemical reactions and produce reactive oxygen species, disrupting cellular processes. To mitigate phototoxicity, researchers use lower laser powers, shorter exposure times, and pulsed lasers to reduce energy delivery. Alternative imaging modalities and the development of less phototoxic fluorophores are also being explored to minimize damage while achieving super-resolution imaging. Furthermore, the development of new fluorophores that are less prone to phototoxicity has been an active area of research.

## 4 Conclusion

Model membranes, such as supported lipid bilayers and giant unilamellar vesicles (GUVs), are simplified systems used to study the properties and behaviors of biological membranes. These models have been crucial for understanding membrane organization, dynamics, and function. However, traditional imaging methods have limitations in resolving the fine details of membrane structures.

Super-resolution microscopy techniques, including STORM, PALM, and SIM, have dramatically enhanced our ability to visualize and study membrane structures at the nanometer scale. These advances have led to several paradigm shifts in our understanding of model membranes.

Considering the old models, lipid rafts were hypothesized to be small, static domains rich in cholesterol and sphingolipids, thought to play key roles in organizing membrane proteins. Super-resolution imaging has revealed that lipid rafts are dynamic, transient nanodomains that can rapidly assemble and disassemble. These techniques have shown that lipid rafts are more heterogeneous in size and composition than previously thought, challenging the notion of uniform raft structures (Owen and Gaus, 2013).

Moreover, proteins were believed to be uniformly distributed in the membrane or organized into large, stable complexes. Super-resolution microscopy has uncovered that membrane proteins are often organized into small, dynamic clusters rather than being uniformly distributed or forming large static complexes. This clustering is influenced by interactions with lipids, other proteins, and the underlying cytoskeleton, highlighting a more complex and dynamic organization than previously understood (Stone et al., 2017; Paparelli et al., 2016).

The fluid mosaic model proposed a relatively homogeneous and fluid membrane where lipids and proteins diffuse freely. Super-resolution techniques have shown that membrane fluidity is highly heterogeneous, with regions of varying fluidity coexisting within the same membrane. This heterogeneity affects how lipids and proteins move and interact, indicating a more complex landscape of membrane dynamics (Sezgin, 2017).

Another important aspect in older models is that membrane curvature was primarily understood in the context of simple geometric models, with a limited understanding of how curvature is generated and maintained at the nanoscale. Super-resolution imaging has provided detailed views of how proteins and lipids contribute to membrane curvature at the nanometer scale. These studies have revealed that specific proteins can induce curvature by clustering into small domains, leading to a more nuanced understanding of membrane topology (Matthaeus et al., 2022).

The role of the cytoskeleton in membrane organization was recognized, but details were limited by imaging resolution. Super-resolution techniques have elucidated how the cytoskeleton interacts with the membrane to organize and segregate nanodomains. These interactions are critical for the spatial and temporal regulation of membrane components, demonstrating a tightly coordinated relationship between the membrane and the cytoskeleton (Mueller et al., 2011).

Finally, functional implications of membrane organization were often inferred from indirect evidence due to imaging limitations. Super-resolution microscopy has enabled direct observation of how changes in membrane organization affect cellular processes such as signaling, trafficking, and membrane fusion. This has led to a deeper understanding of the functional roles of membrane nanodomains and how their disruption can lead to disease (Bartosova et al., 2021; Bartosova et al., 2020).

Super-resolution techniques have significantly advanced our understanding of model membranes, revealing a complex and dynamic landscape that challenges traditional models. These discoveries underscore the importance of high-resolution imaging in uncovering the intricate details of membrane organization and function, paving the way for new insights into cellular processes and potential therapeutic targets.

In conclusion, while super-resolution microscopy techniques have revolutionized biological imaging, they are not without challenges. Future advancements in fluorophore engineering, imaging modalities, and imaging protocols are expected to further enhance the capabilities of super-resolution microscopy and overcome these challenges.

Super-resolution microscopy generates large, high-resolution datasets, but analyzing and interpreting them is challenging due to the need for advanced algorithms and a deep understanding of biological processes. Key challenges include sample preparation, compatibility with live-cell imaging, and quantitative analysis. Specific sample preparation techniques can introduce artifacts, and some super-resolution methods are incompatible with live-cell imaging, limiting real-time observations. Quantifying protein distribution and dynamics within nanodomains is difficult due to cellular complexity and current analytical limitations. Integrating super-resolution data with other techniques is essential for understanding protein organization in plasma membrane nanodomains and its impact on cellular processes, which could lead to new therapeutic strategies.

Addressing these challenges will not only expand our understanding of protein organization within plasma membrane nanodomains and its cellular impacts but also push the boundaries of super-resolution microscopy as a tool for biological research.
